# Enhancing patient safety through the quality assured use of a low-tech video interpreting system to overcome language barriers in healthcare settings

**DOI:** 10.1007/s00508-020-01806-7

**Published:** 2021-02-02

**Authors:** Maria Kletečka-Pulker, Sabine Parrag, Klara Doppler, Sabine Völkl-Kernstock, Michael Wagner, Thomas Wenzel

**Affiliations:** 1 Institute for Ethics and Law in Medicine, University of Vienna, Spitalgasse 2–4, Hof 2.8, Vienna, Austria; 2 Department of Child and Youth Psychiatry, Medical University of Vienna, Vienna, Austria; 3 Department of Pediatrics and Adolescent Medicine, Medical University Vienna, Vienna, Austria; 4 Department of Psychiatry, Medical University of Vienna, Vienna, Austria

**Keywords:** Patient safety, Quality improvement, Digitalization, Remote interpreting

## Abstract

Professional interpretation and translation are key issues in the improvement of public health and patient safety in an area of increased migration and multicultural healthcare system needs. Patient safety requires clear and reliable communication to avoid errors in diagnosis, treatment, and neglect of informed consent. Due to the range of languages to be covered, telephone and video interpretation (VI) can be expected to face up to the demands for trained interpreters available on short notice and in more remote healthcare sites.

In a pilot project, we implemented a new up to date model video interpretation unit and used a mixed methods approach integrating quantitative and qualitative data in assessing barriers encountered prior to the use of the pilot system and satisfaction with the use of video interpretation in a number of clinical settings, including inpatient and outpatient units, in Austria. Of all respondents (*n* = 144) 71% reported frequently encountering language barriers, only 37% reported the use of professional interpreters, 81% reported using siblings, parents or other non-professional interpreters, while a considerable percentage (66%) reported using gestures or drawings to communicate, resulting in very low overall satisfaction rate (only 12%) with the prior situation.

In the qualitative study the users observed rapid availability, data protection compliance, ability to see the interpreter despite physical distance, absence of potential external influence resulting from personal relationships, user-friendly nature of the technique, legal certainty, absence of the requirement for personal presence, and cost savings as key benefits in the use of the new technology. Of the users of the system 88% (*n* = 58) rated it as very good (72%) or good (16%).

## Introduction

The increasing role of language barriers due to migration, multicultural societies, and new refugee groups represents a challenge for healthcare professionals and institutions [1], not only from a medical viewpoint but in terms of liability risks as the duty to provide correct communication rests with healthcare professionals or institutions. Rather than relying on lay interpreters, family members [2–4], or the use of professional interpreters on site, the use of telephone and video interpreting systems have been proposed as alternatives [2]. Both are easily available due to recent developments in low-cost technology and high band width.

### Why video interpreting (VI)?

The negative consequences of language barriers in the public healthcare system are apparent. A patient’s command of language determines help seeking [5], ability to orientate within the healthcare system, and thus to gain access to medical resources [6–8]. This results in unequal access to healthcare, low patient satisfaction [9–11] and makes the quality of medical treatment highly dependent upon linguistic capabilities [12–16]. The situation further represents a dangerous risk and challenge for quality assurance and patient safety [17]. The conversation between patient and healthcare professional suffers most from language barriers.

Betancourt et al. [18] cited the three most common sources of adverse events for foreign language patients: First, the use of unqualified language mediators (relatives and staff, etc.) [19–21], second, the influence of cultural beliefs and traditions on care [22] and third, the misconception of employees to master the foreign language well enough (“get by”) [20, 22].

The negative impact of communication barriers on the importance of shared decision-making in patient safety has been recently demonstrated by Barton in a study with a large sample of rheumatoid arthritis patients [23]. Considering the importance of the doctor-patient conversation as a diagnostic and therapy tool [24–26], the ramifications can be serious, especially in highly dangerous outcomes [27]. It is harder for patients who do not speak the local language to provide adequate informed consent, make treatment decisions, follow further instructions, and manage follow-up dates [28]. Especially in emergency situations, lay interpreters such as hospital employees and family members are employed. This carries risks, including confidentiality, misunderstandings with possibly severe medical consequences and an inadequate emotional load on children and other ad hoc interpreters [1, 4, 29, 30].

The medical consultation is the basis for the formation of informed consent. A language barrier can therefore hinder gaining informed consent which is not only a prerequisite for a medical indication, but also for lawful treatment [31, 32]. The right to informed consent as a foundation for proper treatment is anchored in Article 3 of the World Health Organization (WHO) Declaration on the Promotion of Patients’ Rights in Europe [33], Article 6 of the Universal Declaration, the Biomedicine Convention of the Council of Europe on Bioethics and Human Rights [34], as well as in Article 8 of the European Convention of Human Rights (ECHR) [35] and Article 3 of the Charter of Fundamental Rights of the European Union (CFR) [36]. There is, compared to other countries such as the USA, however, no explicit right to medical consultation in one’s native language, although the consultation must allow the patient to make an informed, self-determined decision. The burden of proof that the patient has understood the information communicated lies with the treating healthcare professional and/or the hospital [16]. Where language barriers prevent informed consent, the healthcare professional is not obliged to conclude a treatment contract and risks legal consequences. The only exceptions to this are medical emergencies [37].

Diagnosing and treating patients with language barriers involves higher costs. This should not guide safety considerations, but costs can be significantly reduced by professional interpreting services. After evaluating 4146 hospital stays in an American emergency room Hampers and McNulty concluded that drawing on the services of a professional interpreter represented the lowest cost burden and resulted in shorter hospital stays, reduced administration of unnecessary medication, and appropriate treatment [38]. Remote interpretation methods can reduce other inefficiencies, such as traveling and scheduling [39]. Furthermore, interpreting visual clues such as gestures, pointing, or facial expressions can help overcome cultural barriers, which makes it the primary reason why patients prefer video interpreting to telephone interpreting in the few studies published [40, 41].

## Patients, material and methods

In many hospitals in Germany, Austria and Switzerland the use of VIs is now considered or has been implemented either on a pilot level or on a continuous base. Still, nearly no research-based data have been published to compare actual needs and present practice with the practical feasibility and user satisfaction. In Austria the Federal Ministry of Health therefore initiated a first pilot project on the topic of quality assurance in the treatment of non-German speaking patients evaluating video remote interpreting (VRI) in public healthcare. The research project evaluated the satisfaction, perceived benefits and problems of healthcare professionals in a dichotomous two-step approach first with existing interpretation strategies and with the intervention, namely video interpreting with a professional interpreter.

To determine the level of satisfaction, strategies used until the present, benefits and problems, the following research questions were formulated:

1. To what extent do healthcare professionals see themselves confronted with language barriers?
2. To what extent are language-based communication problems seen/viewed as influencing treatment situations from the subjective point of the healthcare professionals?
3. Which solution strategies are at present applied to overcome language barriers, and what is the subjective satisfaction level of the healthcare professionals?

As part of the project, we further implemented a new professional VI system with safe online links to improve reliable communication and explored the satisfaction with the new work setting in treating migrants. This focused specifically on the questions of use, impact on daily practice and acceptance by end-users in a group of pilot locations including a range of typical inpatient and outpatient settings in Austrian hospitals.

### Methods

A two-step mixed methods design was used to collect and examine the relevant research data focusing on prior experience, satisfaction, and experiences during the pilot test. Quantitative questionnaires were completed by the healthcare professionals and the VI software recorded relevant data, such as the time stamp, call duration and language used. This was followed by qualitative semi-structured interviews conducted with the same healthcare professionals. Over the course of the project questionnaires were also sent to the patients, and semi-structured interviews were conducted with the interpreters: the results of these data will not be included in this article, which limits its focus to the healthcare professionals.

### Intervention: video interpreting (VI)

- Establishing a VI unit
  For 16 h a day, professional interpreters were available to the healthcare professionals for interpreting via video conference. The most common foreign languages in Austria at that time (Turkish, Bosnian, Croatian and Serbian) and Austrian sign language were offered. The consecutive interpretation mode was selected for its technical simplicity and affordability.
- Recruitment of interpreters
  The professional interpreters master three areas of competence which are acquired through university level training [42], namely linguistic, cultural, and translational competence. To guarantee these competences, cooperation was established with the University of Vienna’s centre for translation studies to recruit and consequently support the interpreters with appropriate academic training. As Meyer et al. stated communication with ad hoc interpreters seem unproblematic to the observer; however, a closer look discloses miscommunication [29].
- Assistance of interpreters and healthcare professionals
  Healthcare professionals and interpreters were trained in advance, preparing them for interpreting via video, altered proximity-distance relationship and increased situational flexibility. To optimize the interpreters’ skills, specialized training on subject-specific vocabulary and new remote interpreting modus was provided. Apart from the scientific translation aspect, sensitizing and educating the healthcare professionals in transcultural competence was a critical aspect.
  To remedy any potential problems, a supervisory unit was established to manage problematic situations occurring during daily practice. Additionally, a psychotherapist provided a workshop about current medical, and specifically psychiatric questions relating to this treatment setting.
- Selection of participating clinical settings (endpoints)
  A total of 12 endpoints in 6 different settings were selected. The choice of endpoints was based on their technical suitability and willingness to take part. The two main settings were walk-in clinics and hospital wards, the remaining were emergency, psychiatry, rehabilitation and pension assessment settings. Both nurses and medical doctors were enrolled in the study. This enabled collection and analysis of setting-specific VI user behavior.
- Technical information and technical support for the endpoints
  Easy access software was used to access the right interpreter at the right place. The software runs on MS Windows ® based systems and uses a special high standard security (Cisco ®) based platform to transmit information between users.
  A hardware solution was provided to endpoints consisting of a standard, low-cost commercial tablet with a microphone and high-definition video camera which could be deactivated, to offer patients a maximum level of privacy. Local technical units implemented the hardware. The system can be installed on larger desktop units or laptops and permits the transfer between different units.

### Data collection methodology

- Quantitative questionnaires for healthcare professionals
  Quantitative questionnaires were chosen as a data collection method to allow the specific properties or characteristics of the samples to be recorded. The aim was not to make a statement regarding the population [43], but to provide an overview of the characteristics of individual variables. A standardized questionnaire was developed, consisting of open, semi-open and closed questions.
  The aim was to elicit data from all healthcare professionals actively involved in the intervention. Data were collected on language barriers reportedly encountered in daily practice, on the problems that arise when providing care, on the solution strategies applied so far, and how satisfactory these are.
  The four-page questionnaire was used to collect data on the professionals’ initial experiences with VI and subjective observations on and satisfaction with its effects.
  The third part of the questionnaire examined professional interpretation and the use of VI.
  To avoid a nonresponse bias, the questionnaire was designed to be completed in less than 5 min and therefore was mainly based on closed questions.
  At 6 of the 12 endpoints, it was not possible to determine the exact number of healthcare professionals who would participate in advance. Therefore, the number of required questionnaires was estimated at 10–30 per endpoint. A total of 320 questionnaires were distributed, with a response rate of 45%.
- Video call connection data
  A crucial part of the quantitative data collected during this project was the video call connection data. The video conference software allowed the different user’s behavior to be documented. The data included the quantity, time stamp, duration, and chosen language of video calls. This enabled a context-specific setting analysis that also included information to make a reliable distinction between real video calls, namely video calls involving interpretation for a consultation, and non-relevant test calls, used for the data analysis. The necessary feedback questionnaires for this task were completed by the interpreters and used to continuously collect data on the date of the video call, the calling end-user, the time stamps, and any specifics, such as technical problems or other occurrences which the interpreter believed to be significant. No information was recorded with respect to the content of the conversation.
- Qualitative semi-structured interviews
  This method was applied last to gain a deeper understanding of the perceived added value of the intervention, and to investigate the healthcare professionals’ level of satisfaction with the tool. The method of semi-structured interviews combines aspects of the standardized and non-standardized interview, combining clear “yes” or “no”-questions and thematically focused, open questions. This method gave interviewees the option to discuss any aspects they considered important.
  Furthermore, this phase was designed to identify whether the use of VI had any influence on the healthcare professionals’ setting-specific work processes. Interviewees were then questioned about the possible advantages and disadvantages of VI compared to other strategies.

### Analysis plan and category building (coding)

The semi-structured interviews were processed using common theoretical coding [44] and qualitative content analysis [45]. This ensured that the transcribed material was methodically controlled, analyzed, and coded. Codification was first open, then selective, with the aim of defining core categories from the previously processed code [45].

Codes were used as a means of breaking down and then reassessing the text. By developing and modifying the codes, a new order emerged in the interviews and this in turn facilitated the processing. It became easier to compare the empiric material with the activity patterns and conditions [44]. Qualitative content analysis method allowed activity patterns and social realities to be visualized.

The descriptive statistics analysis tool was applied to the quantitatively collected data. It served to calculate different parameters predetermined by the research questions, displaying them as graphics and tables. Descriptive statistical parameters within a questionnaire describe only what applies to the respondents of the questionnaires, which is why they cannot provide statements regarding the whole population. Thus, this analysis tool shows the structure of observations and the representation of this structure in form of tables and graphics [46].

Finally, once the qualitative and quantitative materials had been analysed separately, a mixed analysis was undertaken. The meta-inference, i.e. the comparative viewing of the inferences of both strands, composes the “crucial gain of a mixed-methods study, which does not only contain separate method strands, but also method strands that are analysed specifically in relation to each other” [47].

## Results

The following section presents the results using the same dichotomy as explained above. It evaluates the status quo considering language barriers during the treatment of nonnative speakers, and subsequently outline results of the study.

### Treatment of nonnative speakers prior to the pilot project

**Fig. 1:**
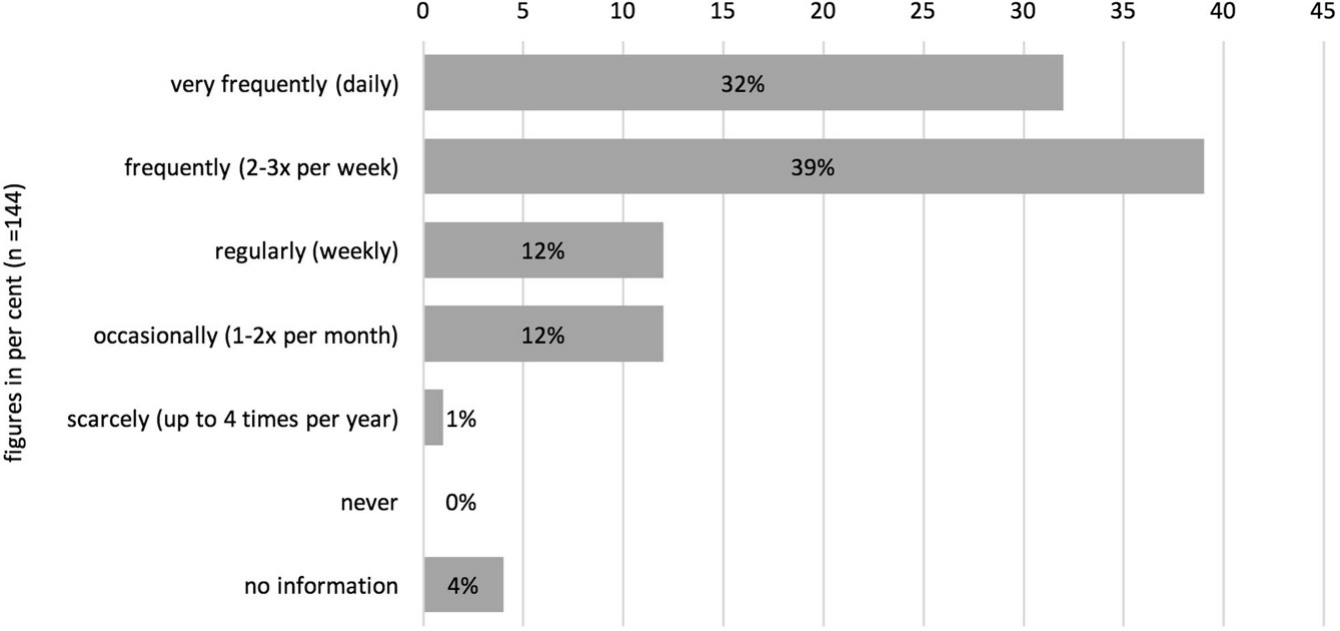
Frequency of occurring language barriers reported by health professionals (*n* = 144)

**Fig. 2:**
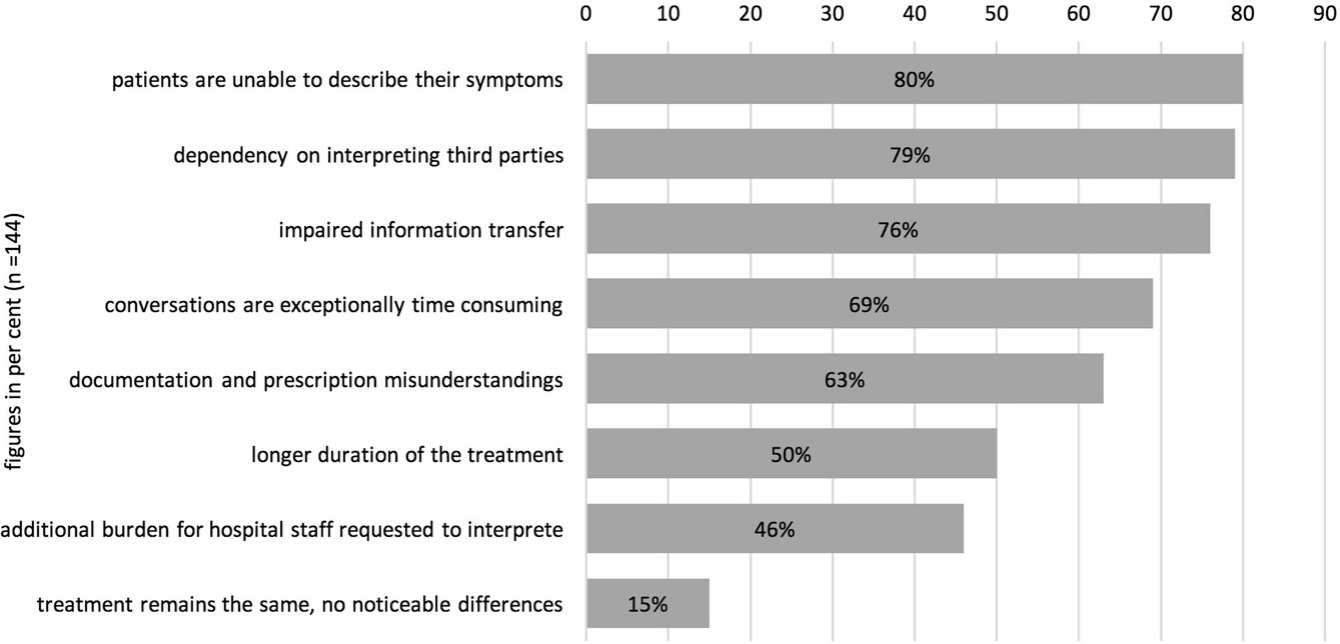
Occurring issues when treating nonnative speakers reported by health professionals (*n* = 144)

- Occurrence of language barriers in day to day medical work
  The questionnaire results indicated that 71% of all respondents (*n* = 144) encounter language barriers at least 2–3 times a week, if not daily (Fig. 1).
  Interviewees repeatedly indicated that the frequency of language barriers is linked to political events, such as labour migration and refugee flows, and accordingly is not constant. This influences the required languages.
- Problems with the treatment of nonnative speakers
  Another subject covered in the questionnaires concerned the most common problems encountered when treating nonnative speakers: 80% of the respondents indicated that patients are unable to properly describe and explain their symptoms which, according to 79% of the respondents caused dependency on third parties for translations; 76% indicated that subsequently it was impossible to properly inform patients about the treatment and possible risks; 69% indicated that the language barrier made it necessary to reduce the pace of the consultation, leading to longer consultation times according to 50% of the respondents and finally, 46% of the respondents experienced language barriers as an additional burden to healthcare professionals, while 15% perceived no differences worth mentioning in the treatment of native and nonnative speakers (Fig. 2).

Of the respondents 78% indicated that conversation in the native language was only possible to a limited extent, while 7% noted having found themselves in situations in which conversation with patients were so problematic that the consultation had to be aborted. In such cases the only possible recourse was to use an acute solution strategy, such as locating a staff member who speaks the relevant language or inviting the patient to return for a new appointment with the request that they bring someone as a lay interpreter. Both solutions can lead to unsatisfactory results, set patient safety at risk and conflict with medical ethics as in the case of children used as interpreters.

### Use of and satisfaction with previously applied solution strategies

Despite the significant difference in quality which exists between professional interpreters and lay interpreters, 95% (*n* = 144) of the respondents indicated that they had previously used a third party to overcome language barriers. The categories of third parties previously relied on consisted of siblings (81%), parents (81%), other family members (81%), friends and acquaintances (71%), general and medical personnel (81%), other patients (42%), internal professional interpretation services (30%), and external professional interpreters (37%).

The majority of used lay interpreters were not employed by the medical establishment for that purpose [25]. Most are family members, friends of the patient, or employees of the medical establishment. The involvement of a child for interpretation can lead to emotional burden and emotional problems for the child [48] and threatens the communication process [4, 19]. As we demonstrated in the pilot project, healthcare professionals often face conflicting interests. They need to maintain the workflow of the medical establishment with no interruptions and delays, whilst sharing the broad consensus among healthcare professionals that this solution is one of the least appropriate and least ethical.

Another solution strategy which creates a clear ethical conflict is the use of other patients as lay interpreters. Of the healthcare professionals 42% indicated that they have used this solution strategy despite wanting to avoid associated problems.

**Fig. 3:**
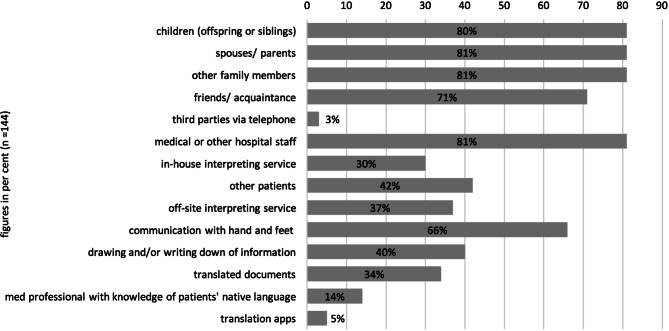
Hitherto existing solution strategies reported by health professionals (*n* = 144)

Other common practise strategies are gestures and postures (60%), use of translated information materials (34%), drawings, and writing down information (Fig. 3).

**Fig. 4:**
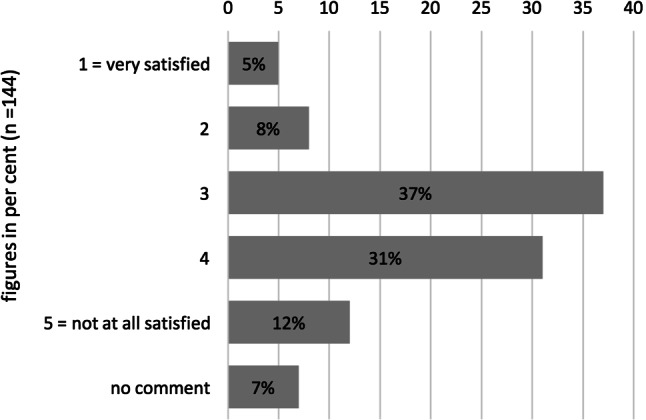
Satisfaction with previous solutions to overcome language barriers in healthcare reported by health professionals (*n* = 144)

Considering the problematic aspects of using non-professional language mediators, it was unsurprising that healthcare professionals tended to answer the question on satisfaction with previous strategies in the negative (Fig. 4).

As the satisfaction level is skewed towards dissatisfaction, there is potential for improvement and room for a different solution strategy. To what extent VI as a solution strategy can fill this gap is analyzed in the second part of this study. The following section addresses the use of this solution strategy and its satisfaction level.

### Video interpreting in the medical setting: numbers and facts from the test phase

- Number, duration and time stamps of video calls
  The 12 participating endpoints undertook 213 video calls during the 6‑month period. The pension assessment centre carried out 54% of these calls, the 4 outpatient departments 15%, the 3 rehabilitation centres 13%, the 2 emergency rooms 11%, the psychiatry ward 6%, and the 3 hospital wards 1% of the calls. The higher level of use in the pension assessment centre reflected their explicit legal duty to provide an interpreter.
  69% of all calls were made between 08:00 and 12:00 and 7% between 15:00 and 22:00. No calls were registered between 06:00 and 07:00, which is due to the end users’ working and consulting hours.
  At 20–22 min, the average call duration was highest at the pension assessment centre, the rehabilitation centre and the psychiatry practice. The outpatient departments and emergency rooms had an average call duration of 10–11 min. The average on wards was 4 min, the average total was 18min.
  The use successively increased monthly except for the final month, namely March 2014. The highest number of video calls (63 calls) were made in February. Use increased as the healthcare professionals gained familiarity with VI, and positive experiences were made.
- Video calls by language
  The languages offered during the project, namely Bosnian, Croatian, Serbian (44%) and Turkish (47%), were almost evenly used. Sign language was used to a lesser extent (9%). During the latter phase, the interviewees indicated a need for other languages including Russian, Czech, Polish, Albanian and Hungarian.

### Video interpreting—an appropriate tool for the healthcare sector?

**Fig. 5:**
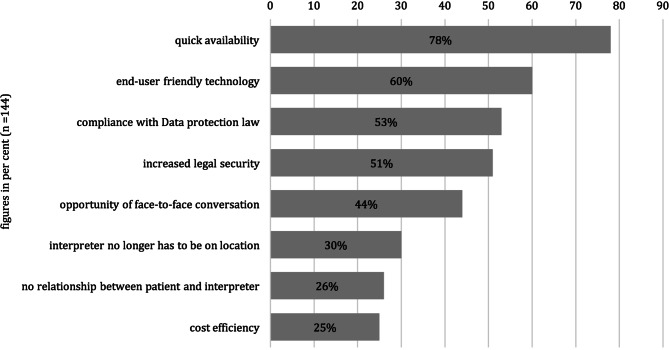
Importance of various aspects associated with the use of video remote interpreting reported by health professionals (*n* = 144)

The healthcare professionals’ personal impressions of key aspects of the VI solution were collated. They identified the following as crucial: rapid availability, data protection compliance, the ability to see the interpreter, the absence of potential external influence resulting from personal relationships, the user-friendly technique, the legal certainty, the absence of the requirement for personal presence, and cost savings. This is congruent with the problems experienced when treating nonnative speakers noted above (Fig. 5).

From 144 healthcare professionals surveyed, at the time of the questionnaire 40% (58 persons) had used the VI tool at least once. When asked if the VI solution was helpful, 88% of those who had already used the system (*n* = 58) rated it as very good (72%) or good (16%), which correlates with the advantages of VI identified above.

Few users rated it as mediocre (5%), unsatisfactory (3%) or bad (2%). One respondent (2%) did not answer.

## Discussion

### The study

First, we documented the low satisfaction with previous methods that are based on inadequate strategies.

Second, we focused on VI with professional interpreters. This part of the study demonstrated that at least on the level of professional interpretation this strategy had a major impact. It became clear that the intervention, quickly and easily accessing professional interpreters through video calls, supported the work of healthcare professionals and relieved staff of additional workload.

Furthermore, the lack of physical presence did not prove a hindrance to language mediation, underscoring the logistical and cost advantages of VI. The study also suggested that, while the duration of the consultations decreased, practitioners perceived a subjective increase in quality.

Introducing this tool involved changes to existing routines and structures. As a result, active involvement of the management, and particularly quality management, are prerequisites for successful long-term implementation. Some pre-existing management issues could hinder the successful implementation of VI. A difference in satisfaction levels could also be observed correlating with the logistical and technical competence of the endpoint.

### Strengths and limitations of this study

Strengths of the study lie in the mixed methods approach, and the inclusion of different medical settings within the sample, to accurately interpret and attribute potentially different outcomes. Only around 30 healthcare professionals attended the original preparation workshops. This was remedied by intense and personal introductions given by the interpreters during first use.

Resource limitations meant that even during the study design the intended study duration of 1 year would not be feasible. As end-users needed to first familiarize themselves with the method, its overall use was lower than expected. Another obstacle was the limited selection of languages on offer, as many more were needed.

It was not possible to monitor technical implementation at endpoints. As a result, successful implementation was dependent on the local technical services, and on support from the commercial company supplying the solution. Therefore, potential influence of researchers on the results was reduced to a minimum.

Limited availability of certain language interpreters, such as Turkish, who met the required professional and academic criteria was problematic. This could be an issue for large-scale application and academic training for languages such as Urdu may be non-existent.

This study focused on the healthcare professionals’ view, and has allowed a diversified and specific analysis of data and influencing factors.

### Outlook

Although healthcare professionals in this study noted that language barriers often occur and have stated their willingness to use VI, the long-term impact on healthcare efficiency, satisfaction and economy needs to be examined.

According to the WHO, the right to language mediation is embedded within the right to informed consent [49], and to equal access to treatment.

As the pilot project in this study shows, once interpretation units are set up and healthcare professionals familiarized themselves with this solution, they are willing to use the technology. User friendliness and easy access need to be considered. This can be expected to benefit all aspects of patient safety, quality of medical care, and equal access to healthcare for patients with different language backgrounds.
